# Layer‐Specific BTX‐A Delivery to the Gastric Muscularis Achieves Effective Weight Control and Metabolic Improvement

**DOI:** 10.1002/advs.202300822

**Published:** 2023-08-08

**Authors:** Siqi Wang, Yuqiong Wang, Long Lin, Zongjie Li, Fengyi Liu, Long Zhu, Jie Chen, Nianrong Zhang, Xinyu Cao, Sunman Ran, Genzheng Liu, Peng Gao, Weiliang Sun, Liang Peng, Jian Zhuang, Hua Meng

**Affiliations:** ^1^ Department of General Surgery and Obesity and Metabolic Disease Center China–Japan Friendship Hospital Beijing 100029 China; ^2^ Department of Mechanical and Automation Engineering The Chinese University of Hongkong Hongkong 999077 China; ^3^ School of Biological Science and Medical Engineering Beihang University Beijing 100191 China; ^4^ Engineering College of Peking University Peking university Beijing 100029 China; ^5^ School of Mechanical and Electrical Engineering Beijing University of Chemical Technology Beijing 100029 China; ^6^ Shanghai Veterinary Research Institute Chinese Academy of Agricultural Science Shanghai 200241 China; ^7^ Department of Ultrasound China–Japan Friendship Hospital Beijing 100029 China; ^8^ Department of Clinical Laboratory China–Japan Friendship Hospital Beijing 100029 China; ^9^ Institute of Clinical Medical Sciences China–Japan Friendship Hospital Beijing 100029 China

**Keywords:** botulinum neurotoxin A, glucagon‐like peptide‐1, microneedle patch, obesity, precise delivery

## Abstract

The rising incidence of health‐endangering obesity constantly calls for more effective treatments. Gastric intramural injection of botulinum neurotoxin A (BTX‐A) as a new modality carries great promise yet inconsistent therapeutic efficacy. A layer‐specific delivery strategy enabled by dissolving microneedles is hence pioneered to investigate the working site of BTX‐A and the resulting therapeutic effects. The drug‐loaded tips of the layer‐specific gastric paralysis microneedles (LGP‐MN) rapidly release and achieve uniform distribution of BTX‐A within the designated gastric wall layers. In an obesity rat model, the LGP‐MNs not only prove safer than conventional injection, but also demonstrate consistently better therapeutic effects with muscular layer delivery, including 16.23% weight loss (3.06‐fold enhancement from conventional injection), 55.20% slower gastric emptying rate, improved liver steatosis, lowered blood lipids, and healthier gut microbiota. Further hormonal study reveals that the elevated production of stomach‐derived glucagon‐like peptide‐1 due to the muscularis‐targeting LGP‐MN treatment is an important contributor to its unique glucose tolerance‐improving effect. This study provides clear indication of the gastric muscularis as the most favorable working site of BTX‐A for weight loss and metabolic improvement purposes, and meanwhile suggests that the LGP‐MNs could serve as a novel clinical approach to treat obesity and metabolic syndromes.

## Graphical abstract

This study provides clear indication of the gastric muscularis as the most favorable working site of botulinum toxin type A for weight loss and metabolic improvement purposes, and meanwhile suggests that the layer‐specific gastric paralysis microneedles could serve as a novel clinical approach to treat obesity and metabolic syndromes.

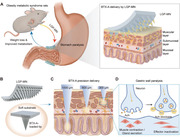

## 1 Introduction

Obesity is one of the major diseases affecting the global population. As a leading risk factor for type 2 diabetes, cardiac disease, stroke, neurodegeneration, and cancer, obesity poses a prime medical challenge and imposes an enormous economic burden on the global healthcare system.^[^ ^1^, ^2^, ^3^, ^4^, ^5^ ^]^ A worrisome consequence is the 2.8 million, and yet increasing, obese‐related deaths occurring each year.^[^ ^6^, ^7^ ^]^ Traditional prescriptions include lifestyle/drug intervention and bariatric surgery (e.g., sleeve gastrectomy and Roux‐en‐Y gastric bypass). However, the former deems only moderate efficacy and weight regain often follows weight loss,^[^ ^8^, ^9^ ^]^ and the latter, which causes permanent body change and possible complications, may not only be intimidating, but also costly to patients.^[^ ^10^, ^11^ ^]^ A minimally invasive and reversible procedure to treat obesity with robust and sustained effects could help tackle this global difficulty.

Neuromodulation has risen in the recent years as a generally minimal invasive strategy to inhibit gastrointestinal motility.^[^ ^12^, ^13^, ^14^, ^15^ ^]^ Botulinum neurotoxin A (BTX‐A) is known as one of the most effective muscle contraction inhibitors, acting by interfering with the release of acetylcholine (Ach).^[^ ^16^, ^17^ ^]^ It enters the cholinergic nerve terminals through binding to and being endocytosed by the botulinum toxin receptors,^[^ ^18^, ^19^ ^]^ and cleaves the SNARE (soluble *N*‐ethyl‐maleimide–sensitive factor attachment protein receptor) complex proteins that mediate the exocytosis of Ach‐loaded synaptic vesicles.^[^ ^20^ ^]^ The inhibition of the release of the cholinergic neurotransmitter aborts the excitatory postsynaptic potential, resulting in local inactivation of the effector cells, such as smooth muscle cells and gland cells.^[^ ^21^, ^22^ ^]^ The neurogenic gastrointestinal events, responsible for initiating major motor patterns and regulating the amplitude of contractions, are mostly dependent on the excitatory neurotransmitter Ach.^[^ ^23^, ^24^ ^]^ Therefore, the BTX‐A‐induced absence of Ach in the stomach should largely abolish gastric motility, including phasic contractions and the resulting peristaltic waves,^[^ ^25^, ^26^ ^]^ which are associated with the rate of gastric emptying. The effect of BTX‐A is specific and reversible, with no significant side effects. It has been reported that the local injection of BTX‐A reduces gastric wall muscle contraction and thus decelerates gastric emptying.^[^ ^23^, ^27^ ^]^ However, the weight loss effects induced by BTX‐A injection vary greatly from case to case.^[^ ^28^, ^29^ ^]^ The inconsistent therapeutic effect may be attributed to the uncontrolled injection depth and uneven drug distribution in the gastric wall, since the three layers of the gastric wall (muscular, submucosal, and mucosal layers), with different structures and functions, may respond differently to BTX‐A. Therefore, to fully exploit the effect of BTX‐A in inducing weight loss after gastric administration, an even and precise delivery strategy is desired to study its neuroinhibitory effect in each gastric layer.

**Figure 1:**
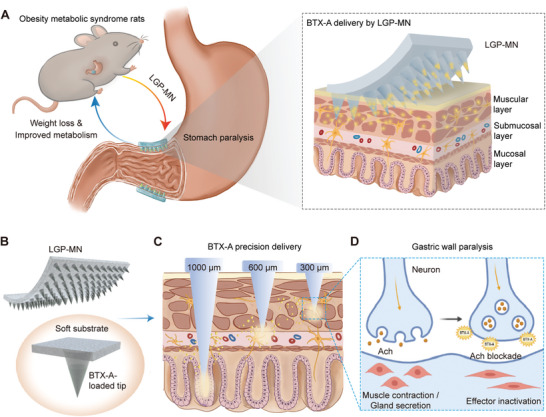
Schematic diagram of the application and mechanism of the layer‐specific gastric paralysis microneedles (LGP‐MNs). A) The dissolving LGP‐MNs deliver BTX‐A into the gastric wall, exhibiting weight loss and metabolism‐improving effects. B) The LGP‐MNs use the two‐step casting technique to fabricate the soft‐substrate dissolving MN patch with refined drug‐loaded tips. C) The LGP‐MNs are designed with a series of heights, separately targeting the muscular (300 µm), submucosal (600 µm), and mucosal (1000 µm) layers. D) Upon MN dissolution, BTX‐A distributes across the designated gastric wall layers and elicits its anticholinergic effect, inhibiting muscle contraction and gland secretion.

In this work, we present a set of dissolving microneedles (MNs), named layer‐specific gastric paralysis microneedles (LGP‐MNs), for the precision delivery of BTX‐A into the three distinctive gastric layers separately.^[^ ^30^ ^]^ The advantages of MNs include targeted drug delivery, minimally invasive and painless administration, and low manufacturing costs.^[^ ^31^, ^32^, ^33^ ^]^ The optimized LGP‐MNs are able to deliver BTX‐A uniformly to the designated layers of the gastric wall through rapid MN dissolution and achieve high working concentrations locally. With the help of the layer‐specific delivery strategy, we are able to reveal the differential therapeutic effects of BTX‐A delivered into the three gastric wall layers. Application on an obese rat model demonstrates that BTX‐A delivery by LGP‐MN to the muscular layer or the submucosal layer alone, could effectively control body weight, delay gastric emptying, restrain feeding, and improve fatty liver and gut metabolic health in comparison to conventional BTX‐A injections. Further, we discover an additional glucose tolerance‐improving effect that is unique to the muscular layer‐targeted delivery. In‐depth studies reveal that while ghrelin inhibition may be a contributor to the weight loss effect exhibited by the submucosal delivery of BTX‐A, higher production of stomach‐derived glucagon‐like peptide‐1 (GLP‐1), a stimulator of insulin secretion, is the main reason for the unique beneficial effect of BTX‐A delivered into and only into the gastric muscularis. This functional study enabled by LGP‐MNs could serve as a primary theoretical basis for further clinical applications of BTX‐A. Meanwhile, the LGP‐MN precisely targeting the gastric muscles could greatly improve the current methods of BTX‐A delivery, and thus bear great translational potential (**Figure** **1**).

## 2 Results and Discussion

### 2.1 Fabrication and Characterization of LGP‐MNs

**Figure 2:**
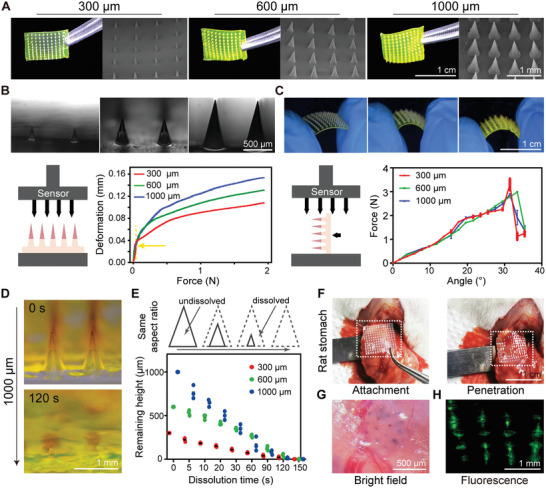
Characterization of LGP‐MN. A) Optical microscopy images and scanning electron microscopy (SEM) images of the three types of LGP‐MN arrays. B) Compression test of the LGP‐MNs. Upper: detailed MN morphology before test. Lower left: schematic diagram of MN subjected to vertical compression by a universal testing machine. Lower right: MN deformation against vertical compressive forces; yellow arrow pointing to the dotted line indicates the minimum force required to penetrate gastric wall tissue. C) Bending test of the LGP‐MNs. Upper: MN patch morphology upon bending. Lower left: schematic diagram of MN subjected to horizontal bending by a universal testing machine. Lower right: bending test results including the break point. D) Representative images of in vitro dissolution of the LGP‐MNs with sodium fluorescein as the model drug. E) Schematic and statistics of in vitro dissolution test of the LGP‐MNs. All three types of LGP‐MNs, with equal aspect ratios, completely dissolve at 120 s. F) In vivo demonstration of LGP‐MN patch application and dissolution. G) Optical microscopy image of the even microwounds left in the gastric wall by the trypan blue‐loaded LGP‐MN tips. H) Fluorescence microscopy image of drug distribution in the gastric wall after dissolution of the sodium fluorescein‐loaded LGP‐MNs.

In order to investigate the possibly distinct weight‐loss effect of BTX‐A in different gastric wall layers, we designed and optimized a set of LGP‐MNs with dissolvable tips for efficient and safe BTX‐A delivery precisely to the designated depth. According to the average thickness of each rat gastric wall layers calculated from cryo‐stat tissue sections, we assigned a series of MN heights (300, 600, and 1000 µm) to different types of LGP‐MNs so that the MN tips insert precisely in the muscular, submucosal, and mucosal layers, respectively (Figure S1, Supporting Information). Each LGP‐MN patch consists of a 1 cm^2^ substrate bearing 10 × 10 conical needles with an aspect ratio of 2 (**Figure** **2**A). The LGP‐MN patches were fabricated with the two‐step casting technique^[^ ^34^, ^35^ ^]^ (Figure S2, Supporting Information).

For better adaptation to the purpose of drug delivery within the gastric wall, sufficient mechanical strength of the LGP‐MN is necessary to ensure its effective penetration into the gastric wall and drug delivery to a specific depth.^[^ ^36^ ^]^ Compression test shows an ~35 µm needle‐tip displacement under a 0.56 N compressive force corresponding to the 3.183 MPa skin‐penetration pressure, which is an unobservable deformation in all three types of LGP‐MNs^[^ ^37^ ^]^ (Figure 2B; Figures S3 and S4A, Supporting Information). The flexibility of the LGP‐MN was tested, and the results showed a maximum flexure of >30% (≈30°), satisfying the surface flexure of the stomach (Figure 2C and Figure S4B, Supporting Information).

We first confirmed the biocompatibility of the LGP‐MN substrate (Figure S5, Supporting Information). The solubility of LGP‐MNs was verified both in vivo and in vitro. In vitro dissolution test revealed the rapid dissolving process of LGP‐MN in contact with phosphate‐buffered saline (PBS, pH = 7.4). All three types of LGP‐MNs completely dissolved within 120 s and all drug‐loaded tips dissolved within 30 s (Figure 2D,E). Similarly, in vivo dissolution test also demonstrated complete dissolution of all three types of LGP‐MNs within 120 s (Figure 2F and Figure S6, Supporting Information). Trypan blue‐loaded and fluorescein sodium‐loaded LGP‐MNs further presented the initial diffusion of payload in the gastric wall in all penetration‐resulted microwounds after MN application (Figure 2G,H). Together, these results demonstrate that the LGP‐MNs are able to achieve sharp penetration, uniform delivery, and rapid dissolution in the gastric wall.

### 2.2 LGP‐MN for Precise Layer‐Specific Drug Delivery in the Gastric Wall

**Figure 3:**
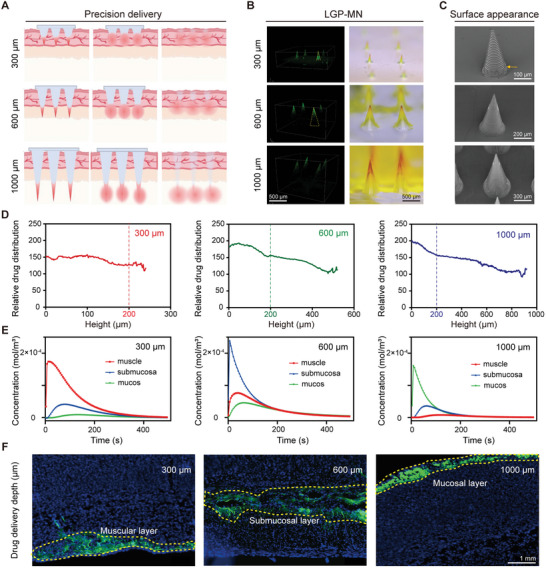
Layer‐specific delivery of the LGP‐MNs. A) Schematic diagram of the layer‐specific precision delivery enabled by the designated heights of the LGP‐MNs. B) Fluorescence microscopy and optical microscopy images showing the sodium fluorescein‐containing LGP‐MN tips (left: green fluorescence; right: red. High concentrations of sodium fluorescein appear red in bright field). C) SEM images of individual MN of the three types of LGP‐MNs. Yellow arrows indicate the bottom of the drug‐loaded region. D) Relative drug distribution throughout the height of the LGP‐MNs calculated through grayscale analysis of the optical microscopy images in (B). The characteristic turning of the curves indicates the concentration of drugs within the 200 µm tip region in all three types of LGP‐MNs. E) Simulated drug distribution in the three gastric layers after drug delivery by the three types of LGP‐MNs. F) Fluorescence images of gastric wall tissue sections after sodium fluorescein delivery by the 300 (left), 600 (middle), and 1000 µm (right) LGP‐MNs.

To study the therapeutic effect of layer‐specific delivery, drugs need to be administered precisely into and distributed evenly within the designated layers of the gastric wall (**Figure** **3**A). Therefore, concentrating the drug within a more refined region of the needle tip is key. We verified both optically and quantitatively the 200 µm drug‐loaded region in the MN tips, leaving a theoretical basis that upon MN dissolution, drugs diffuse and reach maximum working concentration within the layer of delivery (Figure 3B–D and Figure S7, Supporting Information). To investigate the translation of a refined drug‐loaded tip into restrained drug distribution within the desired tissue layer, we first utilized simulation analysis. A primary finite element analysis shows that the drug molecules delivered by the 300, 600, and 1000 µm LGP‐MNs located mainly in the muscular layer, submucosal layer, and mucosal layer, respectively, after the complete dissolution of the MNs (Figure 3E). A more detailed simulation study of drug distribution at the time point corresponding to the peak concentration shown in Figure 3E reveals that the drug molecules concentrated within a very narrow region surrounding the 200 µm MN tip, suggesting precise drug delivery by the LGP‐MNs to the target area (Figure S8, Supporting Information). To verify the simulation results, we prepared a series of sodium fluorescein‐loaded LGP‐MNs with the forementioned heights to investigate the site of drug delivery in vivo. Stomachs were harvested 10 min after LGP‐MN application in the gastric wall, and uniform fluorescence distribution was observed in, and only in the expected layer for each type of the LGP‐MNs (Figure 3F). These results demonstrate that the LGP‐MNs are able to deliver the drug into different layers of the gastric wall with the designated heights, providing a technical basis for the next precision treatment.

### 2.3 BTX‐A Delivery to Different Layers of the Gastric Wall Leads to Distinct Weight‐Loss Effects

**Figure 4:**
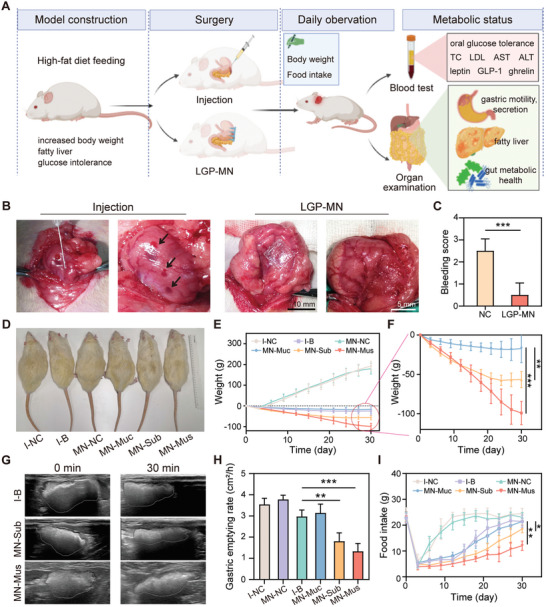
LGP‐MNs induce body weight loss in obese rat model. A) Schematic diagram of the animal experiments. Obesity rat model was established through high‐fat diet feeding, and the rats were treated by BTX‐A injection and the three types of LGP‐MNs, respectively. After the one‐time treatment, the rats were monitored every third day for changes in body weight and food intake. Rats were sacrificed and analyzed for various metabolic status after 1 month of observation. B) Optical micrograph of bleeding from BTX‐A injection versus LGP‐MN treatment. C) Statistics of the number of bleeding sites resulted from injection versus LGP‐MN treatment. D) Representative body size contrast of experimental rats at the end of the observation period. I‐NC, PBS injection; I‐B, BTX‐A injection; MN‐NC, MN delivery of PBS; MN‐Muc, 1000 µm LGP‐MN; MN‐Sub, 600 µm LGP‐MN; MN‐Mus, 300 µm LGP‐MN. E,F) Trends of changes in body weight after different BTX‐A treatments over the 1 month observation period. G) Ultrasound observation of post‐gavage gastric volume changes 2 weeks after BTX‐A treatments. H) Statistical graph of gastric emptying rate assessed through ultrasound. I) Trends of changes in rat food intake over the 1‐month observation period. Data represent mean ± SD; *n* = 7–8. *p < 0.05, **p < 0.01, ***p < 0.001 by Student's *t*‐test.

To evaluate the therapeutic effect of precision delivery by LGP‐MN in vivo, we constructed an obese rat model by feeding high‐fat diet (HFD) for 8 weeks. The resulting model rats showed at least 20% increase in body weight with presence of liver steatosis, impaired glucose tolerance, and elevated aminotransferases (Figure S9A–I, Supporting Information). The rats were then randomly divided into six surgical groups: gastric wall injection with PBS (I‐NC), gastric wall injection with BTX‐A (I‐B), MN delivery of PBS to the gastric wall as a control group (MN‐NC), LGP‐MN delivery of BTX‐A to the mucosal layer (MN‐Muc), LGP‐MN delivery of BTX‐A to the submucosal layer (MN‐Sub), and LGP‐MN delivery of BTX‐A to the muscular layer (MN‐Mus) (**Figure** **4**A). The LGP‐MNs were pressed onto the gastric wall for treatment delivery, which resulted in barely observable bleeding in contrast to the bleeding‐prone syringe injection, demonstrating improved safety (Figure 4B,C and Figure S10, Supporting Information). The treated rats were then monitored for 30 days; body weight and food intake were measured every 3 days. At the end of the experiment their blood and visceral organs were taken for systematic metabolic evaluation.

Body weight shows a decreasing trend in all BTX‐A treatment groups, where the LGP‐MNs delivered the best performance, with a 16.23% weight loss in the MN‐Mus group and 9.29% in the MN‐Sub group. The weight change in the I‐B group and MN‐Muc groups were merely 4.00% and 2.79%, respectively (Figure 4D–F and Figure S11A,B, Supporting Information). This highlights a 3.06‐ and 1.32‐fold better therapeutic effect of BTX‐A delivery to the muscular and submucosal layers in contrast to the conventional injection method. Gastric emptying rate was assessed through ultrasound after BTX‐A treatments. 30 min after equivalent food gavage, ultrasound observation reveals much smaller change of gastric area in the MN‐Mus and MN‐Sub group rats compared to other groups; the subsequently calculated gastric emptying rate also shows statistical significance from the MN‐Mus and MN‐Sub groups to the I‐B group, with a 55.20% and a 39.31% reduction, respectively (Figure 4G,H). The slower gastric emptying rate leads to a subsequent restrained food intake. On day 30, the food intake by both the MN‐Mus and MN‐Sub group rats, and especially the MN‐Mus ones, remained at a significantly lower level than all other groups—43.88% and 13.43% lower than the I‐B group, to be specific (Figure 4I). Assessment of gut energy absorption shows no significant difference among groups, indicating that the weight loss effect of LGP‐MN is attributed to the depressed food intake consequential to the suppression of gastric motility, rather than altered energy absorption levels (Figure S11C, Supporting Information). Among the different treatment groups, MN delivery of BTX‐A precisely to the gastric muscularis displays the best therapeutic effect.

### 2.4 Therapeutic Effect of LGP‐MN on Systemic Metabolic Disorders in Obese Rats

**Figure 5:**
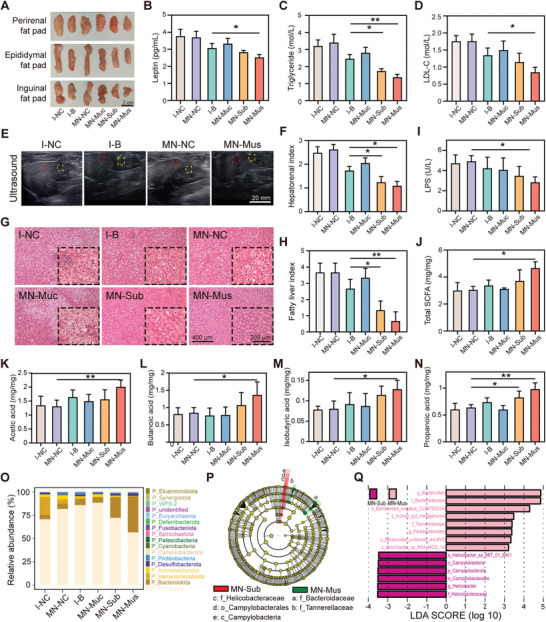
LGP‐MNs ameliorate systematic metabolic disorders. A) Rat fat pads at the end of the animal experiments. B) Plasma leptin levels of each treatment group. C,D) Statistical plots of serum triglycerides levels (C) and low‐density lipoprotein‐cholesterol (LDL‐C) levels (D) after different treatments. E) Representative liver/kidney echogenicity contrast in different treatment groups. Red squares indicate the liver and yellow squares indicate the kidney. F) Rat hepatorenal indices (HRI) based on ultrasound gray scale at the end of the animal experiments. G) Hematoxylin and eosin (H&E) staining of rat livers of different treatment groups. Insets zoom in on the steatotic sites. H) Statistical plots of fatty liver indices reflecting the degree of hepatic steatosis relief after different treatments. I) Circulating lipopolysaccharide (LPS) levels in different treatment groups at the end of the animal experiments. J) Total fecal short chain fatty acid (SCFA) in different treatment groups. K–N) Fecal levels of acetic acid, butanoic acid, isobutyric acid, and propanoic acid. O) Taxonomic analysis of the gut microbiota compositions at the phylum level. P) The LEfSe analysis revealed the most featured bacterial taxa in the gut microbiota. The cladogram represented the discriminative features of the bacterial hierarchy. Q) Histogram of LDA distribution based on LEfSe analysis of categorical information, with LDA score >2. Data represent mean ± SD; *n* = 7–8. *p < 0.05, **p < 0.01 by Student's *t*‐test.

Because obesity is a systemic metabolic disorder, evaluation of liver functions, blood lipids, and gut metabolic health could more comprehensively reflect the change in obese status than monitoring body weight alone.^[^ ^38^, ^39^ ^]^ First, we evaluated the changes in visceral and subcutaneous fat ratios and found a significant decrease after BTX‐A treatment, especially in the MN‐Mus group and MN‐Sub group (**Figure** **5**A and Figure S12A,B, Supporting Information). Measurement of circulating leptin levels using ELISA also demonstrated significantly lowered level in the MN‐Mus group, in line with the fat ratio results (Figure 5B). Blood lipid content indicators such as blood triglyceride levels and low‐density lipoprotein‐cholesterol (LDL‐C) levels also decreased after BTX‐A treatment, with the MN‐Mus group significantly lower than the I‐B group (Figure 5C,D). Then to assess the degree of liver steatosis, we used ultrasound to obtain hepatorenal index (HRI), an indicator of hepatic lipid content depleting individual's renal background. Compared with wild‐type rats, obese rats show elevated HRI, suggesting the appearance of liver steatosis (Figure S9B–E, Supporting Information). After the treatment, the reduced HRI in the BTX‐A‐treated groups suggests the relief of liver steatosis to various degrees, with the most notable improvement in the MN‐Mus group and MN‐Sub group (Figure 5E,F). Further analysis combined with hematoxylin and eosin (H&E) staining revealed significantly smaller intracellular lipid droplets and fewer steatotic cells in the MN‐Mus and MN‐Sub groups compared to the control groups, with the best result in the MN‐Mus group. The fatty liver index, calculated from the H&E staining, decreased by about 75% and 50% in the MN‐Mus group and MN‐Sub group compared with the I‐B group, indicating remarkable relief of liver steatosis after treatment with these two types of LGP‐MNs (Figure 5G,H). Along the line, plasma levels of aspartate aminotransferase (AST) and alanine aminotransferase (ALT) were also found to be significantly attenuated in the MN‐Mus group, suggesting improved hepatocyte damage resulting from liver steatosis (Figure S9H,I and Figure S12C,D, Supporting Information).

We then evaluated two major categories of microbial metabolites, that is, lipopolysaccharide (LPS) and short‐chain fatty acids (SCFA), for the indication of gut metabolic health. HFD and weight gain are associated with elevated circulating LPS level and thus low‐grade systemic inflammation.^[^ ^40^ ^]^ Measurement of circulating LPS levels in different groups of rats shows a significantly lower level of LPS in the MN‐Mus group, indicating that the gastric muscularis‐targeting LGP‐MN treatment could effectively relieve HFD‐induced inflammatory state (Figure 5I). Examination of six common types of SCFA reveals that four types of them, namely, acetic acid, butanoic acid, isobutyric acid, and propanoic acid, are significantly higher in the MN‐Mus group, suggesting potentially improved individual metabolism and immunity after muscularis‐targeting LGP‐MN treatment (Figure 5J–N and Figure S13A,B, Supporting Information). To investigate into the contributing microbial factors, we analyzed the gut microbiota of the rats by sequencing the gut fecal samples using the 16S rRNA gene. High‐quality reads were generated from the merged clean sequences after operational taxonomic units (OTUs) clustering (97% sequence similarity). The predominant microbial communities at the phylum level include Firmicutes (76.07%), Bacteroidota (13.66%), Verrucomicrobiota (4.63%), Actinobacteriota (2.22%), Desulfobacterota (1.62%), and Proteobacteria (1.38%) and others. Previous studies had already proved that obesity was associated with the relative abundances of Firmicutes and Bacteroidetes.^[^ ^41^, ^42^ ^]^ In this study, the ratio of Firmicutes to Bacteroidetes (F/B value) was decreased in the MN‐Mus (3.30%) and MN‐Sub (1.48%) groups compared with the control group (15.44%) (Figure 5O). At the genus level, the relative abundances of *Bacteroides* in all the treatment groups were much higher than the control group, showing the advantage of BTX‐A treatment. Uniquely, the MN‐Mus group presents a higher relative abundance of *Lactobacillus* than all other groups^[^ ^43^, ^44^ ^]^ (Figure S13C, Supporting Information). The linear discriminant analysis effect size (LEfSe) was performed to identify the specific bacterial taxa. The differences in the gut microbiota at different taxon levels were compared and displayed by a cladogram (Figure 5P). The microbiological markers at different phylogenetic levels of the fecal microbiota were also analyzed using the Kruskal–Wallis rank sum test, and the predominant bacteria with linear discriminant analysis (LDA) score >2 were shown (Figure 5Q). A few categories are significantly elevated in the MN‐Mus group. Changes in the compositions of *Bacteroides, Bacteroides vulgatus* CL09T03C04, and *Bacteroides uniformis* dnLKV2 might provide extra health benefits for the host by producing beneficial metabolites, such as the major types of SCFA and other antibacterial compounds. Meanwhile, the *Odoribacter sp* N54MGS14 that is also improved in the MN‐Mus group has recently been reported to regulate the host's innate immune signaling and generate protective immunity.^[^ ^45^ ^]^ Taken together, our results demonstrated that the LGP‐MN delivery of BTX‐A into the muscular layer of the gastric wall leads to the most favorable systematic metabolic disorder improvement.

### 2.5 LGP‐MN Leads to Layer‐Specific Glucose Tolerance Improvement and Gastric Hormonal Changes

**Figure 6:**
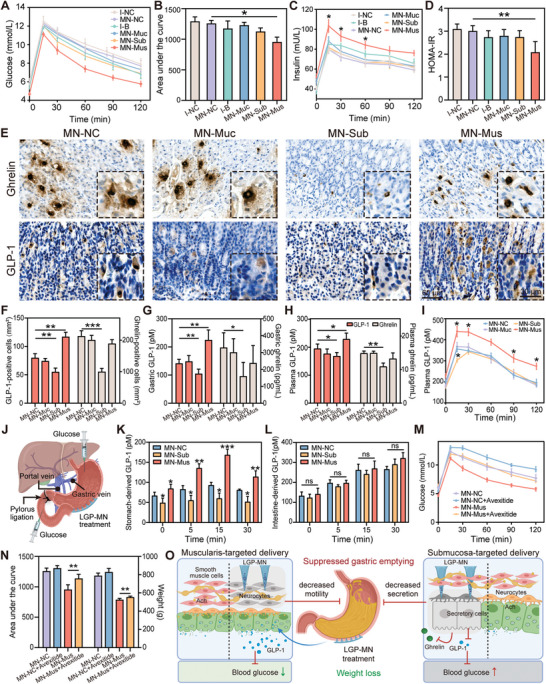
Improvement in glucose tolerance by muscle‐targeting LGP‐MN and possible gastric hormone‐related mechanisms. A,B) Blood glucose levels during the oral glucose tolerance test (OGTT) and analysis of area under the curve (AUC) in each treatment group at the end of the animal experiments. C) Blood insulin levels during the OGTT. D) Homeostatic model assessment of insulin resistance (HOMA‐IR) results of each treatment group at the end of the animal experiments. E) Immunohistochemical (IHC) staining of ghrelin and GLP‐1 in gastric wall tissues. F) Statistics of the number of GLP‐1‐ and ghrelin‐positive cells based on image analysis of the IHC stainings in (E). G) Expression levels of GLP‐1 and ghrelin in the stomach on day 30. H) Plasma levels of GLP‐1 and ghrelin in fasting rats on day 30. I) Plasma GLP‐1 levels during the OGTT. J) Schematic diagram of the pylorus ligation model. 50% glucose was injected from the stomach or the intestine; blood samples were drawn from the gastric vein and the portal vein at each time point. K,L) Stomach‐ (K) and intestine‐derived (L) GLP‐1 levels at 0–30 min after glucose injection. M) Blood glucose levels during the OGTT with and without GLP‐1 receptor antagonist, Avexitide. N) Statistical results of the OGTT AUC and body weight with and without Avexitide. O) Schematic diagram of the possible mechanisms underlying the differential therapeutic effects delivered by the gastric muscle‐ and submucosa‐targeting LGP‐MNs. Data represent mean ± SD; *n* = 7–8. *p < 0.05, **p < 0.01, ***p < 0.001 by Student's *t*‐test.

Since obesity is usually accompanied by a pathological state of abnormal glucose tolerance,^[^ ^46^, ^47^ ^]^ as demonstrated by our obese rat model (Figure S9F,G, Supporting Information), glycemic indices are also monitored to evaluate the therapeutic effect of the LGP‐MNs. To assess the glucose tolerance profile in each group of rats, we performed oral glucose tolerance test (OGTT) after treatments, and the area under the glycemic index curve (AUC) was calculated. Compared with the other treatment groups, only AUC of the MN‐Mus group was significantly reduced (**Figure** **6**A,B). We further measured plasma insulin levels at all corresponding time points and found that, similar to the post‐gavage glucose levels, only that in the MN‐Mus group showed a significant elevation from other groups (Figure 6C). Hence, homeostatic model assessment of insulin resistance (HOMA‐IR) was calculated and, as expected, only the MN‐Mus group showed a significant decrease compared with the control group (Figure 6D and Figure S14A,B, Supporting Information). This suggests that precise muscular paralysis of the gastric wall could improve glucose tolerance at an early stage, which could potentially prevent the worsening of blood glucose and thus relieve diabetic risks. In contrast, BTX‐A delivery in the submucosa did not have the same effect. Combining the previous data, we found that both BTX‐A delivery in the muscular layer and that in the submucosal layer of the gastric wall resulted in slower gastric emptying and significant body weight loss, but only delivery in the muscularis, and not in the submucosa, improved the abnormal glucose tolerance.

To investigate the underlying mechanism, we examined gut‐derived hormones whose levels could be drastically affected by gastric procedures. Ghrelin and GLP‐1 are two gastric hormones that affect metabolism in a weight loss‐independent manner, where ghrelin acts primarily to promote gastric motility, and GLP‐1 inhibits gastric motility and promotes insulin secretion.^[^ ^48^, ^49^ ^]^ Gastric wall tissues were analyzed for ghrelin and GLP‐1 expression levels by immunohistochemical staining and the results were verified on both transcriptional and translational levels (Figure 6E–G and Figure S14C,D, Supporting Information). The results show that the expression of both gastric hormones were significantly suppressed after submucosal delivery of BTX‐A compared to the control group, with 52.83% and 30.94% reduction in the number of ghrelin‐positive and GLP‐1–positive cells, respectively. Interestingly, the number of ghrelin‐positive cells in the MN‐Mus group remained a relatively same level as the control group, yet that of the GLP‐1–positive cells showed a 46.25% increase. Meanwhile, plasma ghrelin and GLP‐1 levels at baseline and after gastric gavage confirmed that the secretion of these hormones in the MN‐Sub and MN‐Mus groups follow the same pattern as their local expression in the gastric wall, suggesting the notable role of stomach‐derived ghrelin and GLP‐1 in circulation (Figure 6H,I). The discrepancy between hormone secretion patterns of these two groups that both demonstrated slowed gastric emptying rate and weight loss effect of the MN treatment may be the explanation for their different performance in improving glucose tolerance. We suppose that the low expression of ghrelin associated with the retardation of gastric motility is the major contributor to the significant weight loss in the MN‐Sub group, and the GLP‐1 level is the deterministic factor for the occurrence of glucose tolerance improvement.^[^ ^50^, ^51^ ^]^

### 2.6 Stomach‐Derived GLP‐1 Mediates Glucose Tolerance Improvement after Muscularis‐Targeting LGP‐MN Treatment

To further investigate into the role of LGP‐MN–responsive GLP‐1 in causing different glucose tolerance behaviors, we adopted the pylorus ligation model to distinguish between stomach‐ and intestine‐derived GLP‐1 (Figure 6J and Figure S14E, Supporting Information). Thirty days after the MN treatments, we ligated the pylorus of the rats, loaded the stomach or small intestine with 2 g of 50% glucose solution per kilogram of body weight, and withdrew blood from the gastric vein or the portal vein with catheter to assess stomach‐ and intestine‐derived GLP‐1 levels, respectively. Results show prominently higher levels of gastric vein GLP‐1 in the MN‐Mus group compared to the MN‐NC control group, yet the MN‐Sub group shows consistently lower levels of stomach‐derived GLP‐1 (Figure 6K). At the same time, the GLP‐1 levels with an intestinal origin show no significant difference between groups (Figure 6L). These results combined indicate that BTX‐A delivery to the gastric muscularis does not affect GLP‐1 release from the small intestine; it results in increased stomach‐derived GLP‐1 release in response to glucose stimulation by upregulating the number of GLP‐1–positive cells in the gastric wall.

Finally, we tested the driving force of GLP‐1 in the metabolic effect of BTX‐A delivery to the gastric muscularis pharmacologically with a GLP‐1 receptor antagonist, Avexitide. The results also show a post‐gavage blood glucose in the MN‐Mus+Avexitide group that is comparable to that of the MN‐NC control group, indicating that the addition of GLP‐1 receptor antagonist cancels the improvement in glucose tolerance brought by BTX‐A delivery to the gastric muscularis (Figure 6M,N). However, the difference in body weight change between the MN‐Mus and MN‐Mus+Avexitide groups suggests that GLP‐1 plays an auxiliary role in the weight loss effect of the muscularis‐targeting LGP‐MN, in which case the major contributor is the BTX‐A–induced muscle paralysis (Figure 6N and Figure S14F, Supporting Information).

Based on the above results, we hypothesize that the therapeutic effects of LGP‐MN observed in different gastric wall layers take separate mechanistic routes. The most direct effect of BTX‐A delivered into the tissue is the inhibition of Ach release from the presynaptic nerve terminals and the abortion of the excitatory postsynaptic potential.^[^ ^18^, ^19^ ^]^ This, in the case of the gastric muscularis‐targeting LGP‐MN, directly leads to muscle paralysis and hence diminished gastric peristalsis. The consequential retardation of gastric emptying elicits an instant effect of reducing food intake, resulting in body weight loss. Meanwhile, the number of GLP‐1–positive cells is increased in the gastric wall, leading to an elevation of stomach‐derived GLP‐1, which improves glucose tolerance while further suppressing gastric emptying and contributing to body weight loss. In contrast, the restrained gastric emptying after the delivery of BTX‐A by LGP‐MN to the gastric submucosa relies more on the inhibition of gland cells following Ach release blockage, where the reduction of ghrelin leads to less robust weight loss, and the reduction of GLP‐1 aborts the glucose tolerance improvement (Figure 6O). Therefore, we propose that BTX‐A delivery into the gastric muscularis induces the best overall therapeutic effect, including weight loss and improved metabolic disorders. Based on the above results, we believe that the LGP‐MN could serve as a safe and effective method to practice the layer‐specific delivery strategy clinically.

## 3 Conclusion

In this study, we developed a novel dissolving LGP‐MN for layer‐specific precision delivery of BTX‐A into the gastric wall. The minimally invasive LGP‐MN largely improved the safety of gastric procedures compared to conventional injection, and was able to distribute drugs rapidly and uniformly in the designated gastric layers. The layer‐specific delivery strategy revealed distinct effects of BTX‐A when administered to different layers. Delivery of BTX‐A to the gastric muscular layer mediated by LGP‐MN demonstrated the most favorable therapeutic effects in an obesity rat model, including weight loss, relief of liver steatosis, healthier gastric microbiota, and improved glucose tolerance. BTX‐A delivery to the gastric submucosa also led to weight loss and improvement of fatty liver, yet the lack of glycemic control effect led us to unveil the underlying role of gastric hormones. The LGP‐MNs allowed us to discover the reduction of ghrelin and GLP‐1 after BTX‐A delivery to the submucosa and the contrary elevation of GLP‐1 after muscular layer delivery, which explained the discrepancy in glucose regulation in the two groups. Combining with endoscopes, the LGP‐MN could serve as a minimally invasive treatment to address obesity clinically. The layer‐specific delivery strategy endows the LGP‐MNs with the potential to deliver other substance (e.g., protein drugs, DNA, etc.) into specific layers of the gastric wall for other relevant diseases, which warrants further investigation.

## 4 Experimental Section

### Materials

Polyvinyl alcohol (PVA) (Mowiol PVA‐203, Mw ≈ 31 000) and polyvinyl pyrrolidone (PVP) (mean molecular weight 58 000, K29‐32) were purchased from Shanghai Aladdin Biochemical Technology Co. BTX‐A (S10970037) was purchased from Lanzhou Biological Preparations Institute Co. Calcein AM, propidium iodide (PI), and Hoechst 33 258 were purchased from Beijing Solaibao Technology Co. All other reagents were analytically pure and could be used without further purification. UV‐sterilized deionized water with a resistivity of 18.2 MΩ cm was used for all experiments.

Vacuum chamber (WIGGENS A510) and blast drying oven (101‐0B) were used to shape and cure LGP‐MN. Optical microscope (JTVMS‐1510T) was used to observe the appearance of LGP‐MN. Motorized displacement‐force test platform (Electroforce 3100) was used to test the mechanical property of LGP‐MN. Fluorescence microscope images were taken using Olympus IX83 inverted fluorescence microscope. Microneedle molds were prepared by microArch S130 3D printer (BMF Material Technology Inc., Shenzhen, China).

### Fabrication of LGP‐MN

All LGP‐MNs were conical with a length‐to‐diameter ratio of 2:1. Three types of LGP‐MN were fabricated: 1) 300 µm height and 150 µm bottom diameter, 2) 600 µm height and 300 µm bottom diameter, and 3) 1000 µm height and 500 µm bottom diameter. The LGP‐MN was prepared as follows:

1) Preparation of the female mold: The resin template of LGP‐MN was printed by high‐precision 3D printer with the accuracy of 2 µm, which was based on the surface projection microstereolithography technology. The first step was to design the model using the software SolidWorks and import the designed model into BMF slicing software for digital processing. To ensure the smoothness of the needle surface, a layer thickness of 5 µm was used in the preparation process. The resin template was placed in a 6‐well plate and polydimethylsiloxane (PDMS) was poured on top. The 6‐well plate was dried in a drying oven at 60 °C for 8 h. After complete drying, the PDMS female mold was removed from the 6‐well plate and the resin template was peeled.
2) Preparation of MN base solution: MN tip solution: 20% PVA and 20% PVP solution were prepared and mixed at a v/v ratio of 1:1. MN substrate solution: 20% PVA, 20% PVP, and 5% HA solutions were mixed at a v/v ratio of 1:1:1. The above solutions were prepared as the standard base and drugs (e.g., BTX‐A, sodium fluorescein, and trypan blue were mixed in as needed). According to the equal mass method, the base solutions of 300, 600, and 1000 µm type LGP‐MNs were diluted one, five, and 40 times, respectively.
3) Molding process: The two‐step casting technique was used to prepare the needle tip and flexible substrate. 33 µL of MN tip solution and 6.7 IU of BTX‐A were thoroughly mixed and added dropwise into the mold. The system was evacuated, and the excess solution on top was scraped off with a spatula. The temperature was controlled at 36 °C for 30 min to fix the drug‐carrying part of the tip into shape. The MN substrate solution was cast on top of the mold, until the solution height reached 4–5 mm. The system was dried at 36 °C for 10–12 h and the MN patch was peeled from the mold and further dried for 5 h for preservation.

### In Vitro LGP‐MN Dissolution

In LGP‐MN dissolution experiment, PBS (pH = 7.4) was added dropwise onto the MN tips and the tip morphology was observed at 10, 20, 60, and 120 s. The morphological changes of LGP‐MN were monitored with an optical microscope.

### Simulation of LGP‐MN Mechanical Properties

Mechanical property simulation of the LGP‐MN was performed using COMSOL Multiphysics 5.5 software. The MN tip was set to be a 15 µm diameter platform. In the tip compression simulation, the bottom of the MN substrate was fixed, the boundary load type was pressure (the resistance of the tissue to the microneedle during the penetration process) applied on the MN tip platform and directed toward the MN substrate, with a pressure value of *P* = 3.183 MPa. In the substrate bending simulation: the boundary load was applied at both ends of the substrate and directed toward the center of the substrate.

### Simulation of In Vivo Drug Diffusion

The diffusion model of LGP‐MN in gastric tissue was based on the porous dilute substance delivery module in the COMSOL Multiphysics 5.5 software. Model dimensions were obtained from experimental measurements: the thickness of gastric serosa was 20 µm, muscular layer 400 µm, submucosal layer 200 µm, and mucosal layer 400 µm; height of the drug‐carrying region was 200 µm in all three types of the LGP‐MN. The main influencing factor during drug diffusion through the MN dissolution in the gastric wall was the effective diffusion coefficient; according to reported mathematical model,^[^ ^52^ ^]^ 5.4999983 × 10^−6^, 5.49999919 × 10^−6^, and 5.49999967 × 10^−6^ cm^2^ s^−1^ were calculated and obtained for the effective diffusion coefficient of BTX‐A in the muscular, submucosal, and mucosal layers of the gastric wall, respectively. The relationship of drug diffusion with time was studied under transient study step.

### Characterization of LGP‐MN Mechanical Properties

1) Microneedle compression performance test: 1) the bottom of the LGP‐MN substrate was fixed to the mobile end (the probe) of the tester; 2) the probe was moved through coarse adjustment until the microneedles were in contact with the fixed platform at the lower end; and 3) the probe was moved downward at a loading speed of 0.1 g s^−1^ until the set load (200 g) was reached.
2) Substrate bending performance test: 1) a side of the LGP‐MN patch was attached to the fixed end of the testing machine so that the MN patch stood vertically; 2) the probe was moved through coarse adjustment until in contact with the top lateral of the LGP‐MN patch; and 3) the probe was moved downward at a speed of 1 mm s^−1^ until the microneedle patch fractured.

### Animal Model and Surgery

All animal experiments were in accordance with the Guide for the Care and Use of Laboratory Animals and approved by the Animal Ethics Committee of the Clinical Research Institute of China–Japan Friendship Hospital. The Veterinary Medical Editors' Consensus of Authors' Guide on Animal Ethics and Welfare were followed during the experiments. Eight weeks‐old male SD rats with a body mass of ≈180–220 g were purchased from Charles River. After 1 week of adaptive feeding, all rats were numbered and alternately assigned to groups using the systematic random grouping method. Among them, the normal control group was fed standard chow and the high‐fat group was fed high‐fat chow. All rat housed in a climate‐controlled facility (12 h light/12 h dark cycle, 20–25 °C) and provided water ad libitum.

After 8 weeks of high‐fat diet feeding, the rats’ body weight and other metabolic indices were assessed to confirm establishment of the obesity model. Animals were randomly divided into the following groups: I‐NC group (*n* = 7), I‐B group (*n* = 7), MN‐NC group (*n* = 7), MN‐Muc group (*n* = 7), MN‐Sub group (*n* = 8), and MN‐Mus group (*n* = 8).

### Surgery

In brief, under inhalation anesthesia with 2% isoflurane, the rat abdomen was sterilized, and a 3–4 cm surgical incision was made in the middle of the abdomen. The stomach wall was exposed and gently wiped with gauze. Injection operation: 0.3 mL of BTX‐A (20 IU) solution was injected, through a 30 G needle, into the gastric wall at multiple points (including sinus, fundus, and body), with 0.1 mL dosage at each point.

### LGP‐MN Delivery Operation

3 LGP‐MN patches were placed at the designated locations on the gastric wall (same as the injection sites) with forceps and pressure was applied immediately to press the MNs into the gastric tissue. After the operation, the abdomen incision was sutured layer by layer and the rat was removed from anesthesia. The entire surgery lasted ≈10 min.

### GLP‐1 Receptor Blocking

After the MN treatment (MN‐NC and MN‐Mus groups), PBS or Avexitide, a GLP‐1 receptor antagonist (Exendin (9‐39), MedChemExpress, Cat# HY‐P0264), was administered through subcutaneous injection to the rats with a dose of 0.727 mg kg^−1^ per day, throughout the 30 days observation period.

### Color Doppler Ultrasound

The gastric emptying rate was measured by Color Doppler Ultrasound 2 weeks after the surgery.

### Ultrasound Operation

Abdominal hair was removed from the anesthetized rat; a small amount of ultrasound gel was applied to the unhaired skin area and the ultrasound probe was moved in the gel‐covered area, and the B‐mode image of the subject's internal organs was recorded. The length, circumference, and area of the stomach were assessed by Color Doppler Ultrasound at *T*_1_ = 0 min, and measured again at *T*_2_ = 30 min, and the gastric emptying rate was calculated for each group. Gastric emptying rate (cm^2^ h^−1^) = (*S_T_*_2_ *_–_ S_T_*_1_)/1 h. Furthermore, HRI was tested. The kidney was localized by identifying the renal artery and cortical/medullary separation. At the same time, the liver was observed in the same view by identifying the homogeneous and smooth contours of the tissue echogenicity. The grayscale pixels of the hepatic and renal echogenicity were calculated and their ratio was obtained as the HRI.^[^ ^53^ ^]^

### Oral Glucose Tolerance and Blood Tests

To perform the OGTT, the rats were given 50% glucose (2 g kg^−1^ body weight) orally after overnight fasting. Afterward, blood glucose was measured at 0, 15, 30, 60, and 120 min.^[^ ^47^ ^]^ Blood glucose levels were measured using a blood glucose meter (LifeScan Inc., Milpitas, CA, USA). Insulin resistance was calculated by using the homeostasis model assessment (HOMA‐IR) index, as defined by the equation

HOMA‐IR = (Fasting Glucose mmol L^−1^ × Fasting Insulin mU L^−1^)/22.5^[^ ^54^ ^]^

### Blood tests

Blood samples were collected in blood collection tubes coated with K2EDTA (BD, Cat# 36 784), and centrifuged for 15 min at 4 °C, 12 000 × *g*. Plasma GLP‐1, ghrelin, leptin, and LPS levels were determined by using corresponding ELISA kits CSB‐E08117r, CSB‐E09816 and CSB‐E07433r, and CSB‐E14247r (CUSABIO), respectively, according to the manufacturer's instructions.

### Calculation of Relative Fat Ratio and Hematological Examination

All rats were closely monitored for 30 days after the treatment, and body weight and food intake were recorded at 9:00 am every 3 days. At the end of the 1 month observation period, the rats were fasted 12 h before sacrifice and sampling, and body weight were measured. After sacrifice, perirenal fat mass, epididymal fat mass, and inguinal fat mass were collected and weighed for relative fat ratio analysis. To reflect visceral fat ratio, relative perirenal and epididymal fat ratio = (left and right perirenal fat mass + left and right epididymal fat mass)/body mass × 100%. To reflect relative subcutaneous fat ratio, relative inguinal fat ratio = left and right inguinal fat mass/body mass × 100%. The collected blood samples were centrifuged at 4 °C, 12 000 × *g* for 15 min and then the serum was separated and tested for triacylglycerol, LDL‐C, insulin, AST, ALT, and NE levels by an automatic biochemical analyzer.

### Pylorus Ligation Model

On day 30 after the MN treatment, the pylorus of rats was ligated with suture, and catheters were inserted into the gastric vein and the portal vein to prepare for blood sampling. Then 50% glucose solution was injected into the stomach or small intestine at a dose of 2 g per kilogram of body weight, and blood was withdrawn through the catheters at 0, 5, 15, and 30 min for GLP‐1 level analysis.

### H&E and Immunohistochemistry

Livers were harvested from sacrificed rats and fixed in 4% paraformaldehyde, dehydrated, followed by paraffin embedding and sectioning at 5 mm thickness. The sections were deparaffinized in xylene and rehydrated in a graded series of ethanol and then subjected to H&E or immunohistochemistry (IHC) staining. For H&E staining, the slides were rehydrated with descending gradient of ethanol, stained with hematoxylin and eosin, respectively, dehydrated with ascending gradient of ethanol, and then cleared with xylene. IHC staining was performed with Vectastain Elite ABC Kit (Vector Laboratories) following the manufacturer's instructions. Citrate buffer (pH 6.0) was used for antigen retrieval, and 0.3% NaHB_4_ was used for immunoperoxidase labeling. Sections were incubated with primary antibody (anti‐GLP1 (Affinity AF0166; Abcam, ab26278), anti‐Ghrelin (Abcam, ab209790)) at 4 °C overnight. Immunoreactive sites were visualized with 3, 30‐DAB and the resulting signals were visualized using a confocal laser scanning microscope (Olympus BX61, Tokyo, Japan).

Evaluation of steatosis in the sections was performed by two experienced pathologists using the NAFLD histological scoring system where the degree of steatosis was graded into four levels. A score of 0 was assigned for <5% steatosis, 1 for 5–33% steatosis, 2 for 34–66% steatosis, and 3 for >66% steatosis.^[^ ^55^ ^]^

### ELISA Assessment of Gastric Hormones

Gastric wall tissue stored in liquid nitrogen was first washed with precooled PBS, and grinded on ice in precooled PBS supplemented with protease and phosphatase inhibitors (Thermo Scientific, Cat# 78 442). The fully grinded tissue was centrifuged at 13 000 rpm, 4 °C, for 20 min. The supernatant was extracted, and protein concentration was assessed using the BCA method (Solarbio, PC0020). ELISA was performed using rat GLP‐1 and ghrelin ELISA kits CSB‐E08117r and CSB‐E09816 (CUSABIO) according to the manufacturer's instructions.

### DNA Extraction and 16S rRNA Gene Amplicon Sequencing

Bacterial DNA was extracted from about 30–50 mg of fecal content using the QIAamp Fast DNA Stool Mini Kit (Qiagen, Valencia, CA, USA). The universal primers 338F (5′‐ACTCCTACGGGAGGCAGCAG‐3′) and 806R (5′‐GGACTACNNGGGTATCTAAT‐3′) were used for polymerase chain reaction (PCR) amplification of the V3 and V4 regions of the bacterial 16S rRNA gene. The amplification program consisted of 1 pre‐denaturation cycle at 95 °C for 3 min, followed by 27 cycles of denaturation at 95 °C for 30 s, annealing at 55 °C for 30 s, extension at 72 °C for 30 s, and finally, 1 extension cycle at 72 °C for 10 min. PCR products were recovered using a DNA Gel Recovery Kit (Axygen Inc, Corning, NY, USA), purified using a DNA gel extraction kit (Tiangen, Beijing, China), and sequenced on an Illumina MiSeq sequencing platform (Illumina, San Diego, CA, USA). After removed failed sequences, the sequences were spliced using FLASH software. Then quality control and data optimization of the splice quality were performed using Trimmomatic software.

### Bioinformatics Analysis

UPARSE (version 7.1) was used to cluster the OTUs with 97% similarity; chimeric sequences were identified and removed. The taxonomy of each OTU representative sequence was analyzed by RDP Classifier (version 2.2) against the Silva 16S rRNA database using confidence threshold of 0.7. The microbial taxonomic compositions of the sampled bacterial communities were implemented using the R package software. The LEfSe was performed to search for the taxa in which the relative abundances were significantly different among the different groups.

### Fecal Bomb Calorimetry

Rats were housed individually in metabolic cages with less padding and feces were collected over a 24 h period. Then feces were dehydrated for 72 h, and heat of combustion was determined using an automatic oxygen bomb calorimeter (Huanuo, ZDHW‐8000A). The sample calory was normalized to dry feces weight. Energy absorption was then calculated according to the equation below: Energy absorbed (cal d^−1^) = Amount of dry food ingested (g d^−1^) × Calories in food (cal g^−1^) – Calories in feces (cal g^−1^).

### SCFA Concentration Determination

Fifty milligrams of fecal sample was collected in a 1.5 mL centrifuge tube, then 0.3 mL of water and 0.05 mL of phosphoric acid solution was mixed in, vortexed and shaken for 15 min. Then 0.5 mL of ethyl acetate was added, and the mixture was vortexed and shaken for 30 min, and centrifuged for 10 min at 12 000 rpm, 4 °C. Fifty microliters of the supernatant was transferred into a 2 mL vial, to be measured. The data were detected on an SH‐PolarD column, and the GC‐MS reanalysis data analysis software was used according to established protocols.

### Statistical Analysis

All statistical analyses were performed using Prism version 8.0 software (GraphPad, CA). Data in this work were presented as the mean ± standard deviation (*n* ≥ 3). NS represents not significant, and statistical significance were labeled by ns for not significant, * for *p* < 0.05, ** for *p* < 0.01, *** for *p* < 0.001, and ns for not significant. Statistical evaluations between two groups were performed by Student's *t*‐test. Experiments with more than three groups were evaluated by one‐way ANOVA followed by Bonferroni's test. Repeated‐measures ANOVA was used for OGTT that involved repeated measurements from the same rat.

## Conflict of Interest

The authors declare no conflict of interest.

## Author Contributions

S.W., Y.W., and L.L. contributed equally to this work. H.M. conceived the platform, gathered funding, and led the project. H.M., J.Z., and L.P. gathered funding and provided guidance with experimental design. S.W., Y.W., and L.L. co‐wrote the paper. L.L., F.L., and L.Z. designed and fabricated the device. S.W., Y.W., Z.L., J.C., and N.Z. designed the animal experiments and analyzed the results. X.C., G.L., and P.G. contributed to data analysis and provided advice on this work.

## Supporting information

Supporting Information

## Data Availability

The data that support the findings of this study are available from the corresponding author upon reasonable request.
