# Supplementary material for: Layer‐Specific BTX‐A Delivery to the Gastric Muscularis Achieves Effective Weight Control and Metabolic Improvement
Source: Adv Sci (Weinh). 2023 Aug 8;10(28):2300822. doi: 10.1002/advs.202300822 (PMC10558648; doi:10.1002/advs.202300822)
Supplement: Supplementary file 1 — Supporting Information [file ADVS-10-2300822-s001.pdf]

ADVANCED SCIENCE
Open Access

Supporting Information

for *Adv. Sci.*, DOI 10.1002/advs.202300822

Layer-Specific BTX-A Delivery to the Gastric Muscularis Achieves Effective Weight Control and Metabolic Improvement

Siqi Wang, Yuqiong Wang, Long Lin, Zongjie Li, Fengyi Liu, Long Zhu, Jie Chen, Nianrong Zhang, Xinyu Cao, Sunman Ran, Genzheng Liu, Peng Gao, Weiliang Sun, Liang Peng, Jian Zhuang* and Hua Meng**

Supplementary information

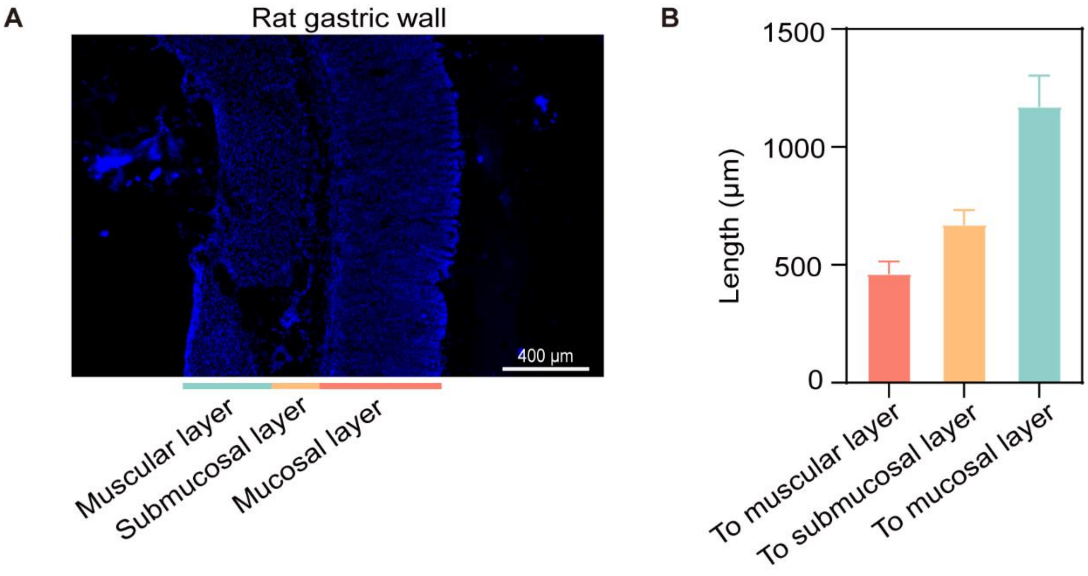

Figure S1. Measurement of rat gastric wall thickness. A) Representative fluorescence microscopy image of the gastric wall cross-section of the obesity rat model. **B)** Distance from the gastric serosa layer to the distal end of the muscular layer, the submucosal layer and the mucosal layer, respectively.

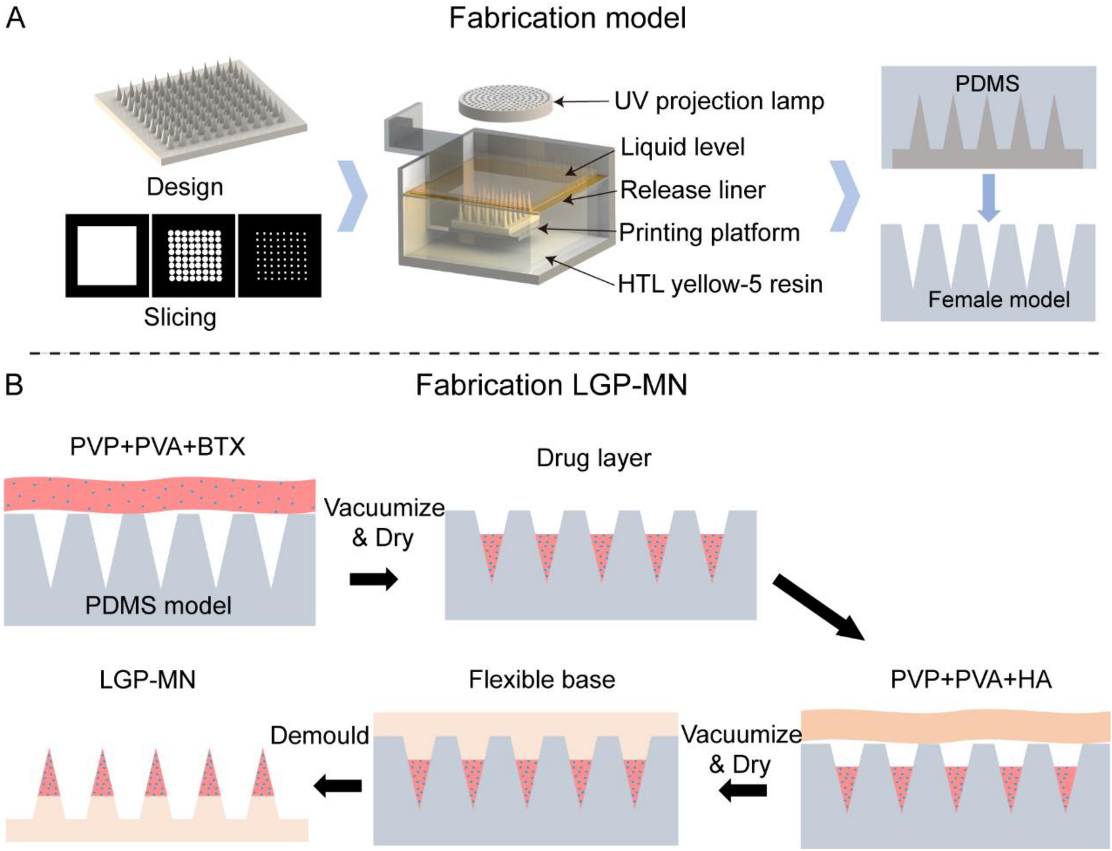

Figure S2. Schematic diagram of LGP-MN fabrication process. A) Preparation of the female mold. The digital model of the LGP-MN resin template is sliced into 5-μm layer diagrams and input into the 3D printer for high-precision printing based on surface projection stereolithography. The female mold is fabricated with PDMS using the resin template. **B)** The preparation of LGP-MNs using the two-step casting technique. The drug-loaded MN tip solution is added into the female mold, evacuated and dried; then the MN substrate solution is cast on top repeating the same steps.

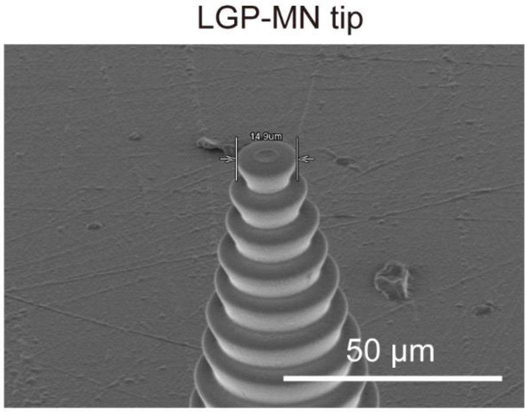

Figure S3. SEM image of LGP-MN tip SEM image with a local magnification on the 14.9-μm wide tip of the 300 μm LGP-MN**.**

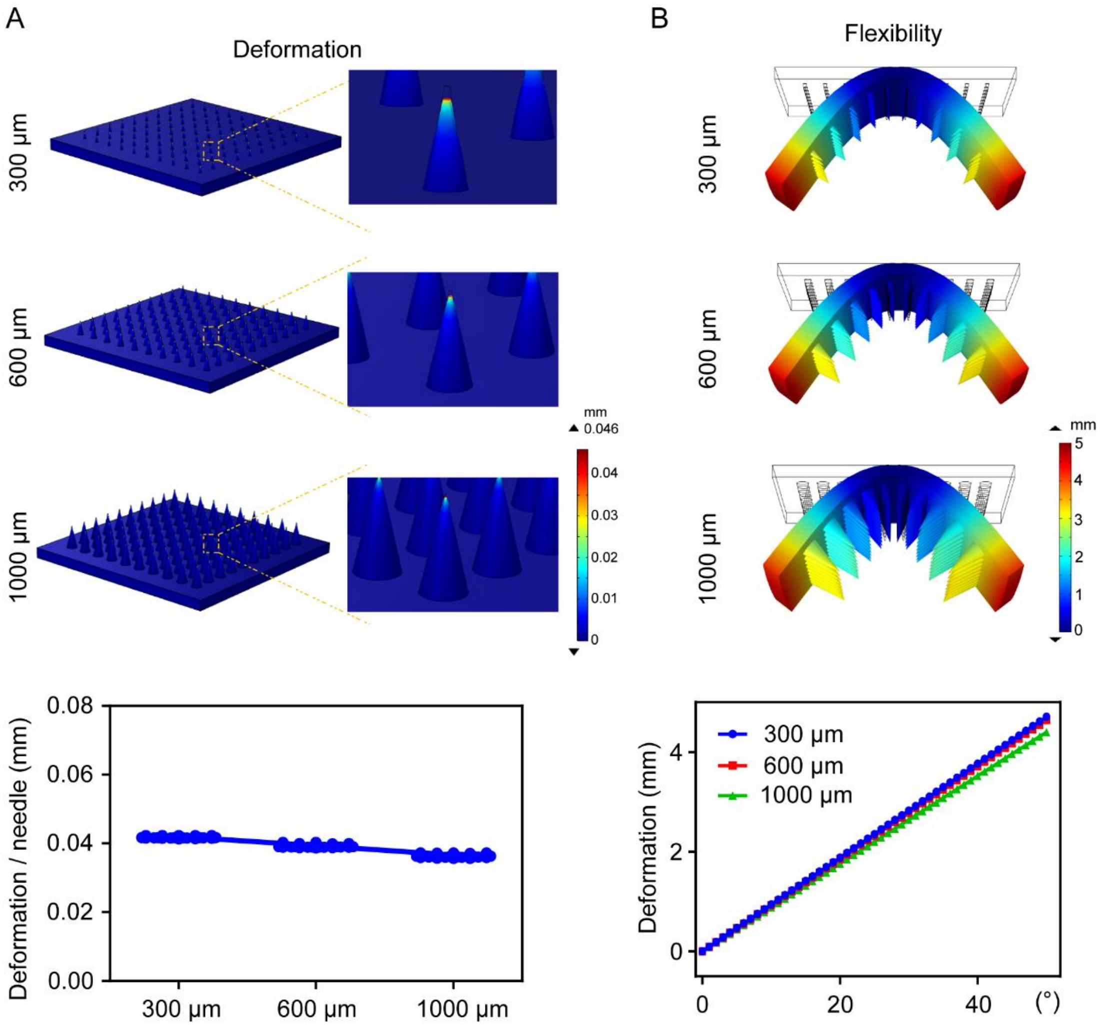

Figure S4. Simulation with solid mechanics parametric model to verify the mechanical properties of LGP-MN. A) Deformation cloud diagram of the whole LGP-MN under an applied vertical pressure of 3.183 MPa (0.056 N). The magnification shows that the maximum deformation occurs at the tip of the LGP-MN and the maximum deformation of all three types of LGP-MNs is ≈ 40 μm. The compressional deformation is independent of the height of different types of LGP-MNs and is proportional to the aspect ratio. **B**) Deformation cloud diagram of the whole LGP-MN under lateral compressive force. The lateral deformation remains a linear relationship with the bending angle before 45° is reached.

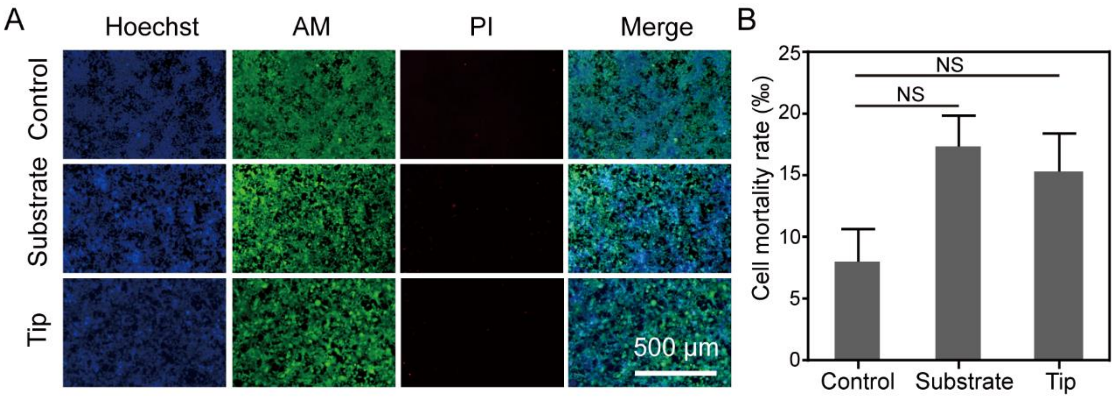

Figure S5. ***In vitro*** **biocompatibility evaluation of LGP-MN base materials. A)** Fluorescence microscopy images of SiHa cells treated with PBS (control), MN substrate material (PVA+PVP+HA) and MN tip material (PVA+PVP). Hoechst 33258 for nuclei (blue); calcein AM for living cells (green); PI for dead cells (red). Scale bar, 200 μm. **B)** Statistical analysis of cell mortality, calculated as the percentage of cells with PI out of all cells.

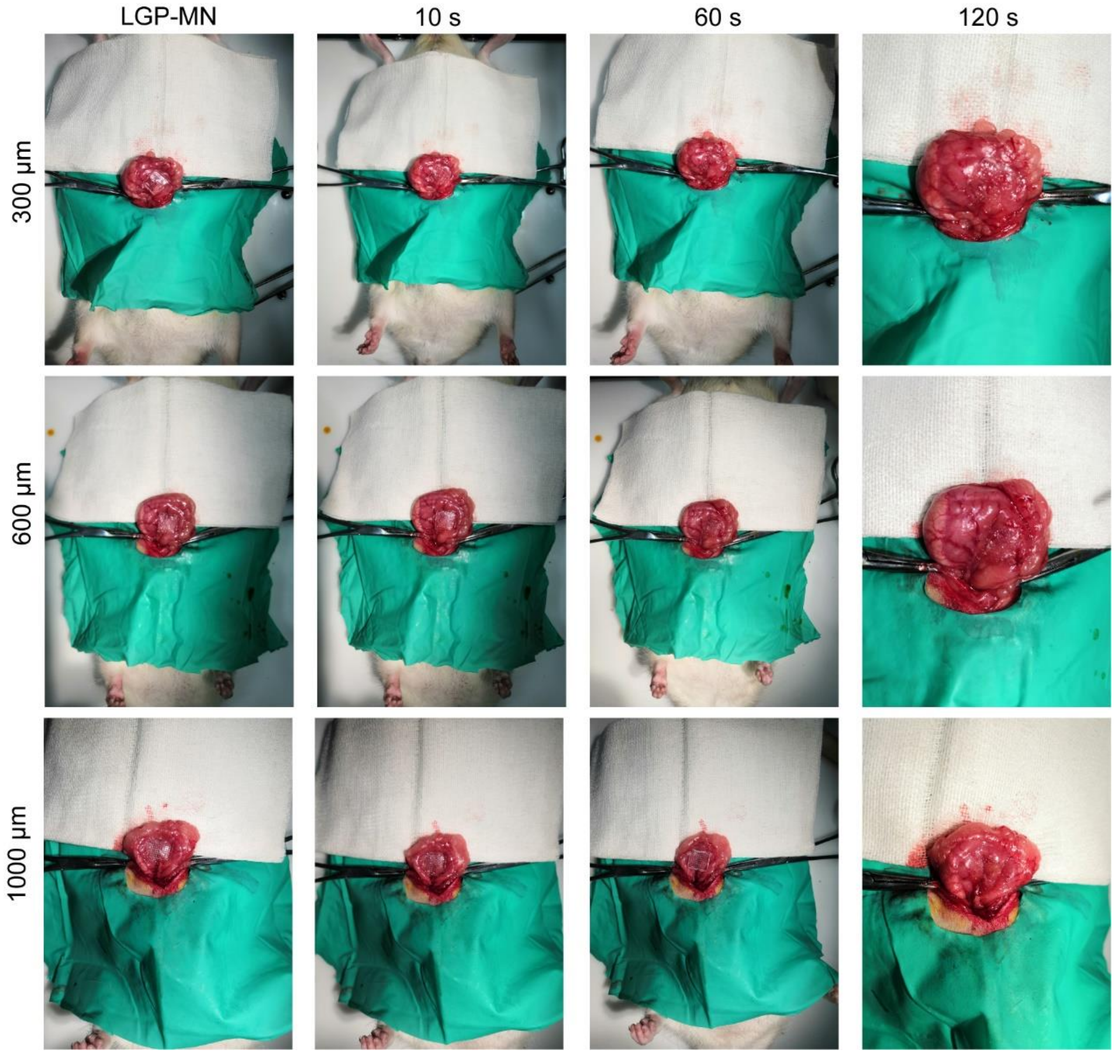

Figure S6. Morphology of the three types of LGP-MNs during the dissolution process. All LGP-MNs, regardless of the different lengths of the MNs (300, 600 and 1000 μm), dissolved within 120 s *in vivo*.

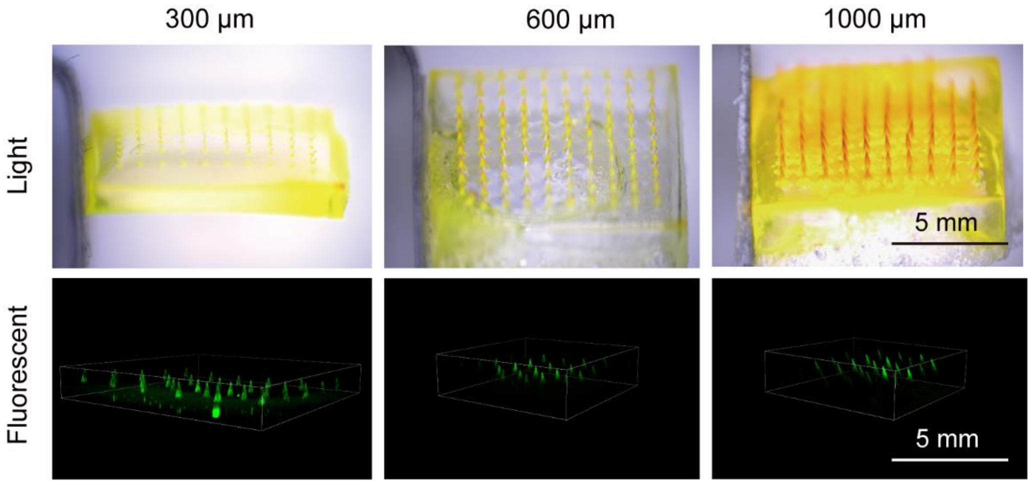

Figure S7. Micrographs of LGP-MN array Optical micrographs and fluorescence micrographs of the three types of LGP-MNs showing the drug-loaded (sodium fluorescein) MN tips**.** (Light: red. High concentrations of sodium fluorescein appear red in bright field. Fluorescence: green fluorescence).

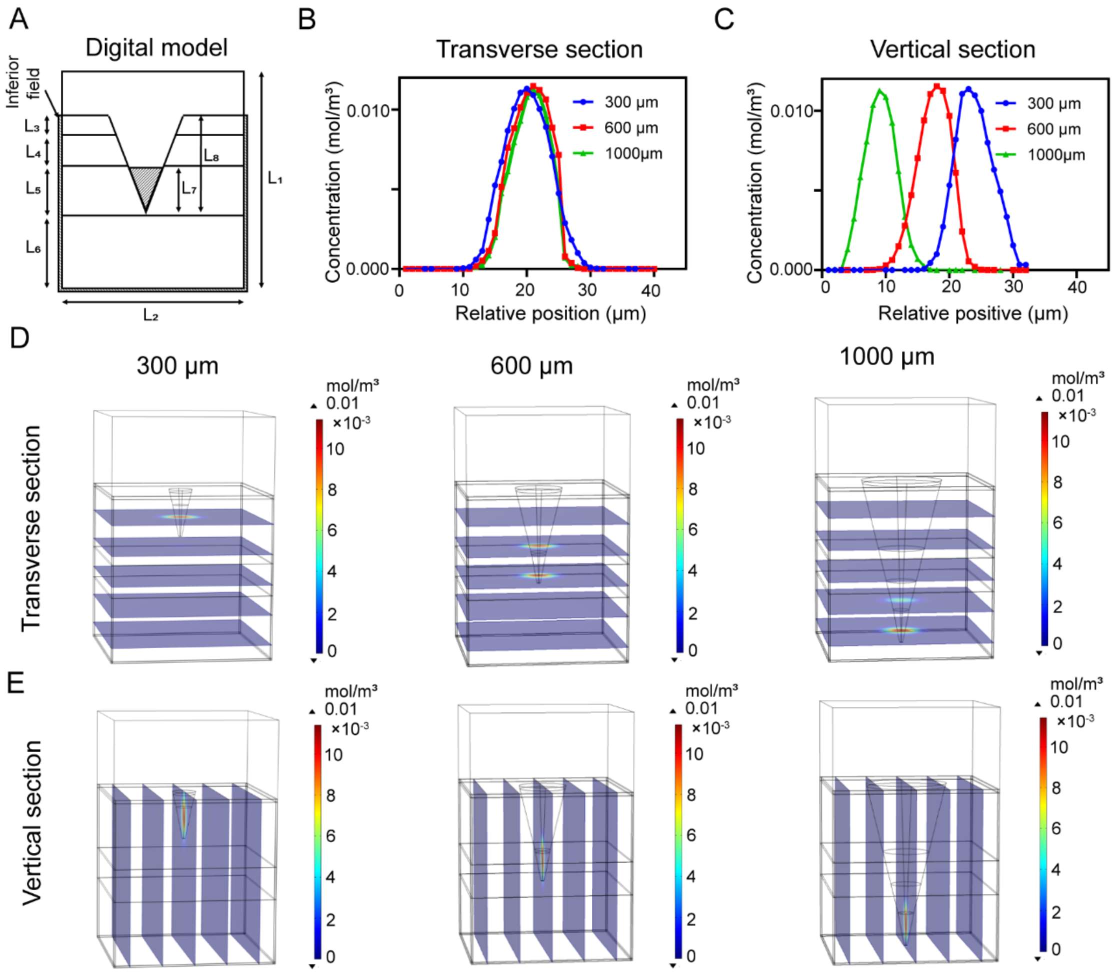

Figure S8. Simulation of drug diffusion from LGP-MN *in vivo*. A) Digital model setup. Simulation is based on a unit model of a single MN in three layers of the gastric wall with infinite boundary field. Parameters of the unit model include: L_1: unit height, L_2: unit width, L_3: gastric serosa layer, L_4: gastric muscular layer, L_5: gastric submucosal layer, L_6: gastric mucosal layer, L_7: drug-containing region, L_8: MN height. **B, C)** Simulated drug (BTX-A) distribution in the transverse section (B) and vertical section (C) of the individual MN after 5 s of MN dissolution. **D, E)** Sectional cloud diagram of drug distribution in transverse (D) and vertical (E) sections of the unit model after 5 s of MN dissolution.

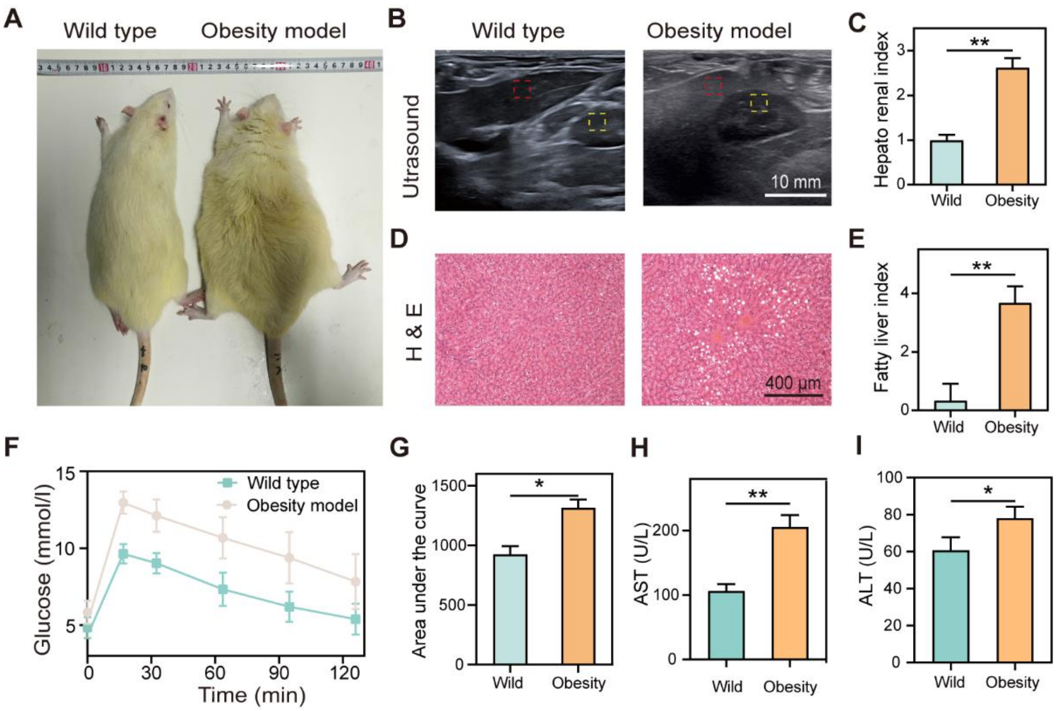

Figure S9. Establishment of obesity model from wild-type SD rats. A) Body size comparison between normal chow-fed rats (wild type) and high-fat chow-fed rats (obesity model) at the end of the 16-week separate diet feeding. **B)** Representative liver/kidney echogenicity contrast in normal chow-fed rats and high-fat chow-fed rats. Red squares indicate the liver and yellow squares indicate the kidney. **C)** Rat hepatorenal indices based on ultrasound gray scale from **B**. **D)** Hematoxylin and eosin (H&E) staining of the livers of normal chow-fed rats and high-fat chow-fed rats. **E)** Fatty liver indices calculated form the H&E staining. **F)** Blood glucose recorded from the oral glucose tolerance test. **G)** Statistics of the area under the glycemic curve. **H, I)** Plasma levels of aspartate aminotransferase (AST) (H) and alanine aminotransferase (ALT) (I) in chow-fed rats and high-fat chow-fed rats.

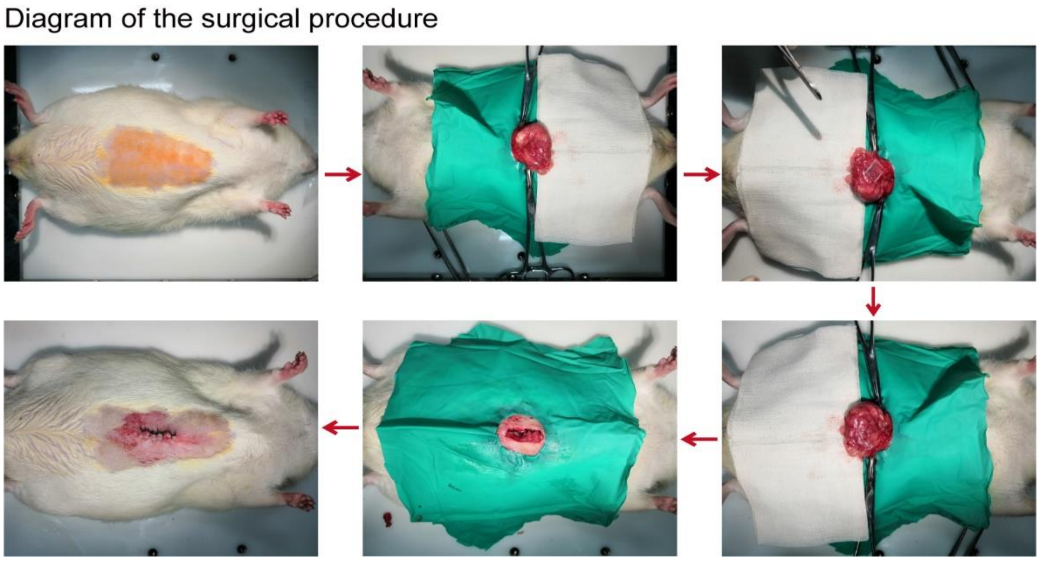

Figure S10. Surgical procedure for LGP-MN treatment. Under inhalation anesthesia, the rat abdomen was sterilized, and a 3-4 cm surgical incision was made in the middle of the abdomen. The stomach wall was exposed and gently wiped with gauze. LGP-MN patch was placed on the gastric wall and immediately pressed to penetrate the gastric tissue. After the operation, the abdomen incision was sutured and the rat was removed from anesthesia.

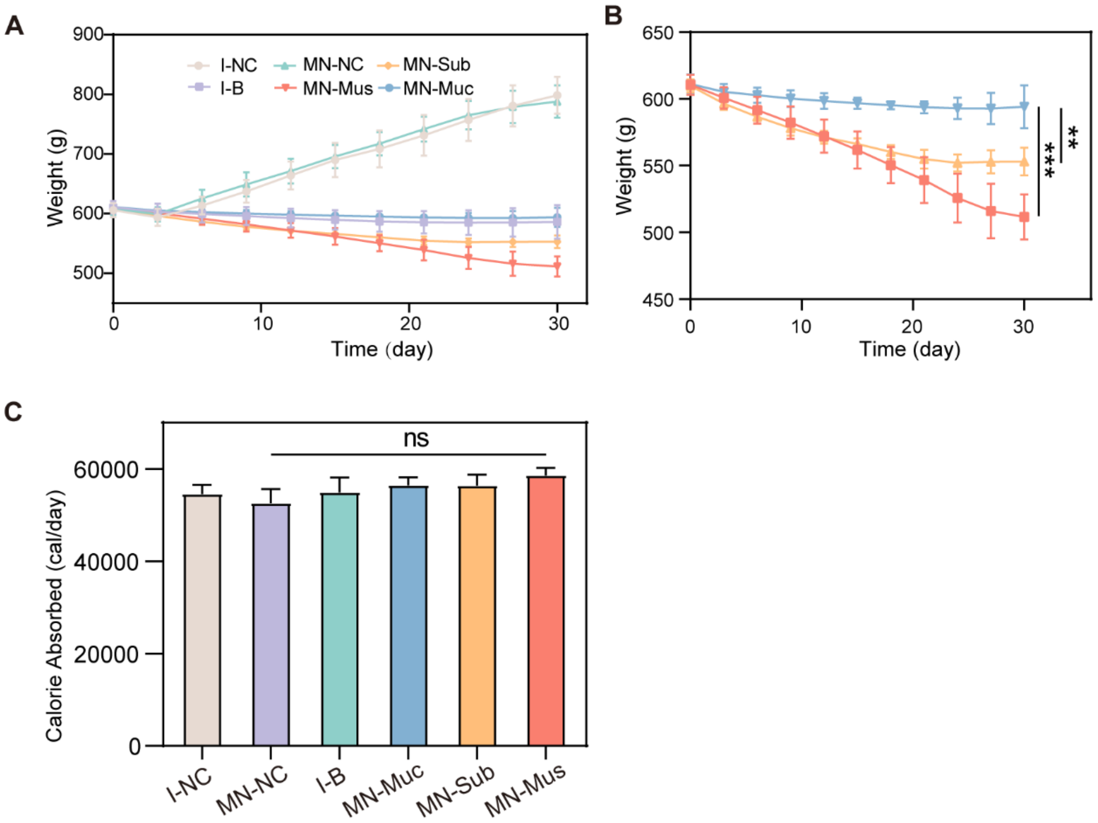

Figure S11. Weight loss and energy absorption. A, B) Changes in absolute body weight after different BTX-A treatments over the one-month observation period. **C)** Energy absorption calculated from the fecal bomb calorimetry test.

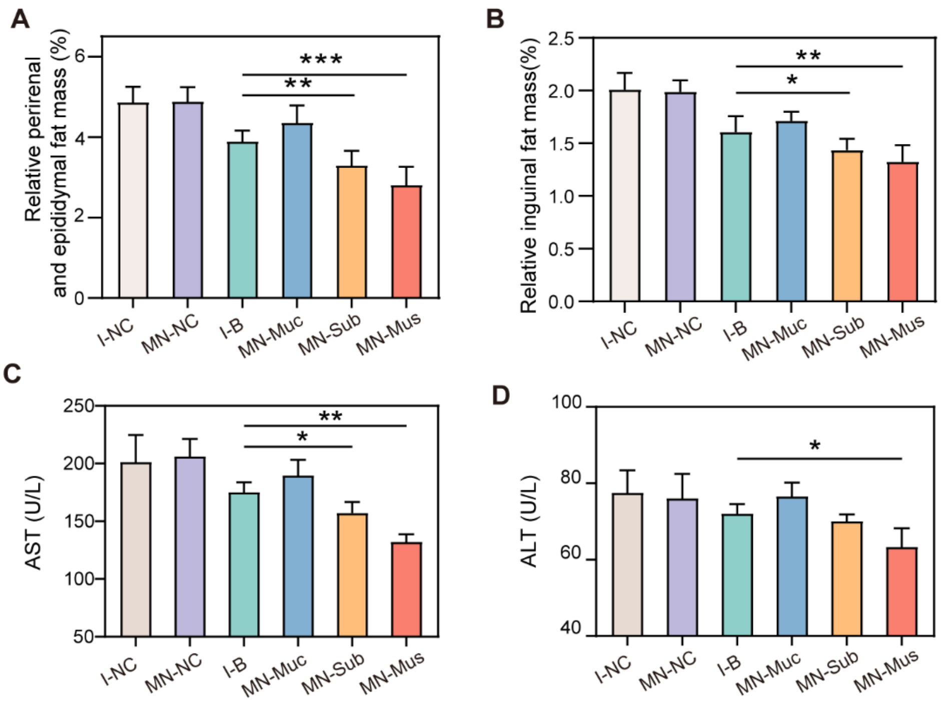

Figure S12. Indices of metabolic disorders in each treatment group. A, B) Statistics of the perirenal and epididymal (A) and inguinal (B) fat content relative to the total body mass. **C, D)** Plasma AST (C) and ALT (D) levels in each treatment group.

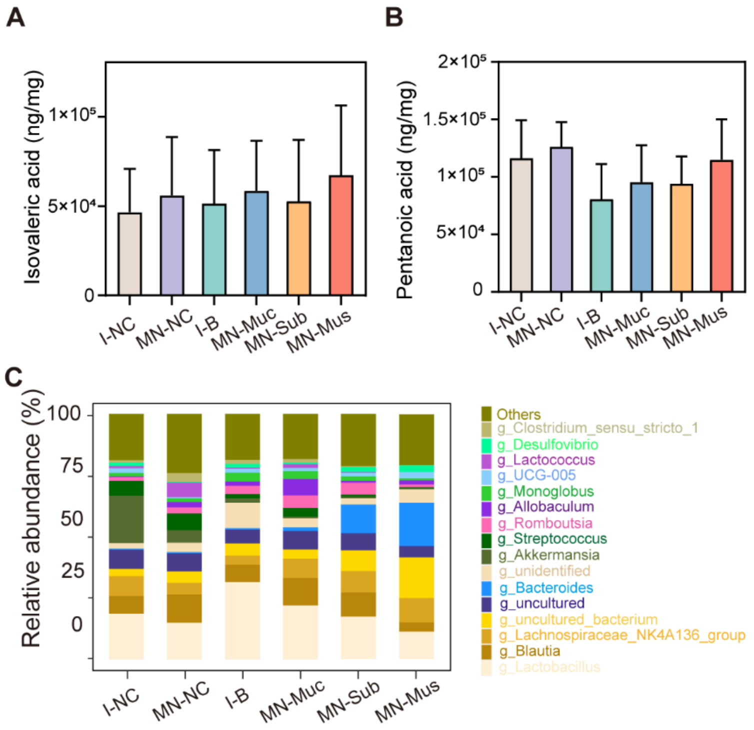

Figure S13. Additional data reflecting gut conditions. A, B) Other two types of common fecal SCFA in different treatment groups. **C)** Taxonomic analysis of the gut microbiota compositions at the genus level.

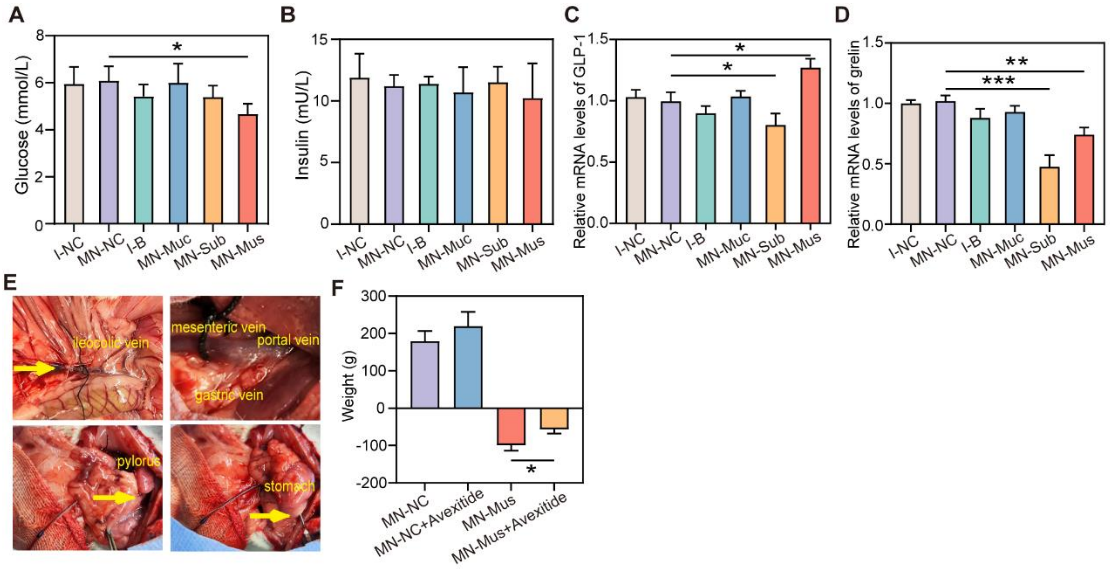

Figure S14. Additional data for the mechanism investigation of the unique glucose tolerance improvement shown in MN-Mus group. A, B) Blood glucose (A) and insulin (B) levels used for the calculation of HOMA-IR. **C, D)** qPCR analysis of GLP-1 (C) and ghrelin (D) mRNA expression. **E)** Photographs of the surgical procedures for pylorus ligation, catheter insertion and glucose injection. **F)** Changes in body weight associated with the use of Avexitide on day 30.

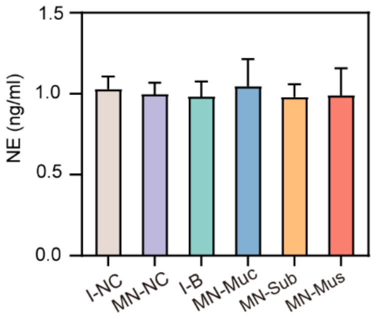

Figure S15. Plasma norepinephrine (NE) levels at the end of the animal experiment.
